# Dietary Supplementation with L-Citrulline Between Days 1 and 60 of Gestation Enhances Embryonic Survival in Lactating Beef Cows

**DOI:** 10.3390/ani15162398

**Published:** 2025-08-15

**Authors:** Kyler R. Gilbreath, Michael Carey Satterfield, Lan Zhou, Fuller W. Bazer, Guoyao Wu

**Affiliations:** 1 Department of Animal Science, Texas A&M University, College Station, TX 77843, USAfuller.bazer@ag.tamu.edu (F.W.B.); 2 Department of Statistics, Texas A&M University, College Station, TX 77843, USA

**Keywords:** arginine, beef cows, citrulline, conceptus, nutrition, pregnancy rate

## Abstract

Low fertility limits reproductive efficiency in cattle. This study was conducted with multiparous Brangus cows receiving dietary supplementation with or without L-citrulline [Cit; an immediate precursor of L-arginine (Arg)]. During the entire experimental period, cows grazed green pasture and had free access to drinking water and mineral blocks. One hundred and seven (107) cows were assigned randomly to one of three treatment groups: dried distillers grains with solubles (DDGS) without Cit supplement (n = 36); DDGS top-dressed with rumen-protected Cit product (RPAA; n = 36); or unprotected Cit product (RUAA; n = 35). After 2 months of lactation, all cows were synchronized to estrus and were bred once via artificial insemination. From Day 1 to Day 60 of gestation, cows were individually fed once daily 0.84 kg of a supplement (DDGS; control), 0.56 kg of DDGS plus 0.28 kg of RUAA (containing 0.07 kg of unencapsulated Cit), or 0.56 kg of DDGS plus 0.28 kg of RPAA (containing 0.07 kg of rumen-protected Cit). The supplemental dose of Cit was equivalent to 0.5% of the estimated daily intake of 14 kg dry matter from pasture. On Days 40 and 60 of gestation, ultrasound was used to determine pregnancy rates. Each pregnant cow had a single conceptus. On Day 60 of gestation, blood samples were obtained from the jugular vein. All cows grazed normally and appeared healthy. Birth rates for live-born calves were 22% and 35% in cows receiving DDGS alone and Cit supplementation, respectively (*p* < 0.05). The beneficial effect of Cit was associated with increases in concentrations of Cit (+19%), Arg (+20%), ornithine (+19%), proline (+17%), and insulin (+82%) but decreases in concentrations of ammonia (–14%) in maternal plasma (*p* < 0.05). Thus, dietary supplementation with Cit is a simple, novel, and cost-effective nutritional method to increase the reproductive efficiency of lactating beef cows.

## Simple Summary

High rates of embryonic deaths are a major factor limiting reproductive efficiency in cattle, particularly in tropical and subtropical regions of the world. However, effective nutritional methods to prevent or alleviate this problem are not available. Results of studies with swine, sheep, rats, and humans revealed an important role of arginine (a conditionally essential amino acid for gestating mammals and their fetuses) in embryonic and fetal survival, growth, and development via the production of bioactive molecules, including nitric oxide, polyamines, and creatine. Because arginine is extensively degraded by ruminal microbes, it must be supplied to ruminants either as a rumen-protected supplement or via intravenous or intragastric administration to impact reproductive performance. An alternative to arginine is the use of citrulline based on our recent discovery that extracellular Cit does not undergo catabolism by ruminal microbes in cattle and sheep. Results from the present study indicate that dietary supplementation with 0.5% citrulline (in either a rumen-protected or unprotected form) to lactating beef cattle between Days 1 and 60 of gestation increased concentrations of citrulline, arginine, and insulin in maternal blood, decreased those of ammonia (a metabolite that is highly toxic to embryos and fetuses at elevated concentrations), and improved embryonic survival. This simple method of dietary supplementation for pregnant cows eliminates the need for encapsulation of citrulline or arginine via expensive engineering techniques and the impractical intravenous or intragastric infusions of citrulline or arginine in production settings.

## 1. Introduction

The greatest limitation to reproductive efficiency across mammalian livestock species is embryonic mortality, which amounts to 25% to 60% [1,2,3]. In particular, the rate of pregnancy loss is estimated to be 30–60% in beef cattle (e.g., 43% for lactating beef cows), with most losses occurring during the first month of gestation [4,5]. Of interest, pregnancy loss can be as high as 80% in some heifers due to genetic and environmental factors [6,7]. The improvement of functional traits using conventional approaches of phenotypic testing and quantitative genetics is difficult, because most reproductive traits are complex (polygenic) with low heritability [7].

Research with swine, sheep, rats, and humans has shown that L-arginine (Arg) is a nutritionally essential amino acid for the growth, development, and survival of the conceptus (embryo/fetus and placenta) [1,8,9,10,11,12,13]. Of note, dietary supplementation with 0.83% Arg to gilts between Days 30 and 114 of gestation [14] or with 1.07% Arg to rats between Days 1 and 21 of gestation [15] increased the number of live-born offspring by 2.0 and 3.4 per litter, respectively. In mammals (including ruminants), Arg can be metabolized to both nitric oxide (NO) and polyamines in maternal and fetal tissues, as well as the uterine–conceptus interface [16,17,18]. These bioactive molecules act directly on the conceptus to activate the mechanistic target of rapamycin (MTOR) cell signaling pathway to stimulate proliferation, migration, and protein synthesis by trophectoderm cells that are essential for elongation of the blastocyst and pregnancy recognition signaling [19,20,21,22,23,24]. Importantly, Arg stimulates the expression of interferon tau in the ruminant conceptus [25]. Besides serving as the pregnancy recognition signal in ruminants, interferon tau acts in concert with progesterone to regulate the expression of a multitude of genes critical to the growth and development of the conceptus, including transporters of nutrients into the uterine lumen [1].

Arg is degraded extensively by ruminal microbes [26,27,28,29,30] and therefore it must be supplied to ruminants either as a rumen-protected supplement [31,32,33,34,35,36,37] or via intravenous or intragastric administration [9,26,29,38,39,40,41,42,43,44] to impact reproductive performance. An alternative to Arg is the use of L-citrulline (Cit; an immediate precursor of Arg). Cit is a neutral amino acid that is converted into Arg via argininosuccinate synthase and lyase at a nearly 100% efficiency in extrahepatic tissues in mammals [45,46,47,48,49,50,51,52] including ruminants [53,54,55]. We recently discovered that extracellular Cit does not undergo catabolism by ruminal microbes in cattle and sheep due to the lack of uptake by the microbes [56,57,58]. This finding has been confirmed in other studies involving sheep [59,60,61,62,63,64,65], goats [66,67], and cattle [68]. Based on the foregoing results, the present study was conducted to test the hypothesis that dietary supplementation with Cit as either a rumen-protected Cit product (RPAA) or an unprotected Cit product (RUAA) would improve embryonic/fetal survival in cattle. Lactating beef cows were chosen for this study because they generally exhibit the highest embryonic/fetal losses among beef cattle [4,5,69].

## 2. Materials and Methods

### 2.1. Animals and Diets

During the entire experimental period, multiparous Brangus lactating cows grazed green pasture and had free access to drinking water and mineral blocks (≥96% NaCl, 1.00% S, 0.15% Fe, 0.25% Zn, 0.30% Mn, 0.009% I, 0.015% Cu, 0.0025% Co, and 0.001% Se; United Salt Corporation, Houston, TX, USA) [58]. One hundred and seven (107) cows were used for this study between 26 May 2016 and 17 March 2017. There were no first- or second- parity cows. The cows were blocked according to body condition score (BCS) and body weight (BW) and were assigned randomly to one of three treatment groups: dried distillers grains with solubles (DDGS) without Cit supplement (n = 36); DDGS top-dressed with RUAA product (n = 35); or DDGS top-dressed with RPAA product (n = 36). No statistical power calculation of sample size was performed, and all beef cows available in our research facility were used in this study. In beef cows, BCS on a 1 to 9 scale is a visual and tactile assessment of their fat reserves, with 1 being extremely thin and 9 being obese [70]. For example, a cow with a BCS of 1 is severely emaciated and physically weak, and has easily visible bones in the shoulder, ribs, back, hooks, and pins; a cow with a BCS of 3 is thin with the deposition of fat in the foreribs, a clear appearance of the last three or more ribs, and highly visible backbones; a cow with a BCS of 5 has moderate fat deposition in the brisket, and her spine and transverse processes cannot be seen; a cow with a BCS of 6 (good condition) has a smooth appearance throughout the body, and her ribs are fully covered with fat and are not noticeable; a cow with a BSC of 8 is obese and has a thick neck, the brisket distended with fat, and an udder with some fat deposition; and a cow with a BCS of 9 is very obese, her bone structures are not easy to identify, and her udder has substantial fat deposition.

**Figure 1:**
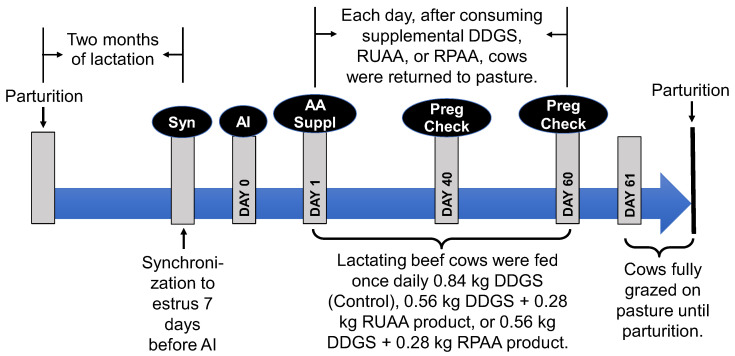
Timeline of the experiment. After 2 months of lactation, all beef cows were synchronized to estrus (Syn), followed (one week later) by artificial insemination (AI; Day 0 of gestation). One day after the AI services, cows in the control [dried distillers grains with solubles (DDGS) only], rumen-protected citrulline (RPAA), and unprotected citrulline (RUAA)] groups were fed their respective supplements once daily before grazing pasture for 60 days. Pregnancy (Preg) was checked on Days 40 and 60 of gestation for each cow using the transrectal ultrasonography method. Between Day 61 of gestation and parturition, cows fully grazed on pasture without any DDGS or citrulline (AA) supplementation. After the end of the 60-day period of AA supplementation, calves from the previous pregnancy continued to stay with their mothers until they were weaned at 6 months of age.

After 2 months of lactation, all cows were synchronized to estrus as described by Williams et al. [71], with modifications. Briefly, cattle were restrained in a hydraulic squeeze chute. On Day 1, a Controlled Internal Drug Release (CIDR) insert (providing 1.38 g of progesterone; Zoetis, Parsippany, NJ, USA) was inserted into the vagina of each cow. On Day 7, the progesterone insert was removed and the cows received an intramuscular injection of 5 mL of prostaglandin F_2α_ (25 mg). Twelve hours after observed estrus, each cow was bred via artificial insemination (AI) with 0.5 mL of semen (the day of breeding = Day 0 of gestation) by the same technician. Each cow received AI only once. The characteristics of cows in the three treatment groups on the day of AI are summarized in Table 1, and the values for all the cows (n = 107) were as follows: days postpartum, 67.5 ± 1.0 days; age, 6.23 ± 0.27 years; BW, 463.4 ± 7.4 kg; and BCS, 4.56 ± 0.08 (mean ± SEM). Cows were assigned randomly to one of two pastures with the similar content of nutrients (Table 2), which were analyzed as we described previously [57]. The timeline of the experiment is highlighted in Figure 1.

An extensive clinical exam of the reproductive tract was not performed before AI to identify reproductive problems. All the cattle had successfully calved in the prior calving season and no issues were encountered upon palpation of the cervix and during AI. Therefore, the likelihood of extensive prior reproductive problems impacting the study was minimal.

One day after breeding until Day 60 of gestation, cows were individually fed daily 0.84 kg of a supplement (DDGS; control), 0.56 kg of DDGS plus 0.28 kg of RUAA (0.07 kg of Cit plus 0.07 kg of L-glutamine plus 0.14 kg of soybean hydrogenated oil in their unencapsulated forms), or 0.56 kg of DDGS plus 0.28 kg of RPAA (0.07 kg of Cit plus 0.07 kg of L-glutamine plus 0.14 kg of soybean hydrogenated oil in their rumen-protected forms). The supplemental dose of Cit was equivalent to 0.5% of the estimated daily feed intake (14 kg dry matter) of a cow on pasture. Both RUAA and RPAA were obtained from Biotechnology Services and Consulting, Inc. (Coppell, TX, USA). The ratio of DDGS to an AA supplement product was 2:1 to facilitate consumption by the cows. DDGS was selected as a supplement because it is readily available and commonly used in beef cattle operations [72,73]. On each day of the 60-day supplementation period, cows were brought from pasture to a pen, separated from their calves, and individually fed their respective supplements once daily. Immediately after the supplement was consumed, cows along with their calves were returned to their original pasture. The first 2 months of gestation were chosen for supplementation because most pregnancy losses in beef cattle occur during this period [4,5,69].

On Days 40 and 60 of gestation, pregnancy was determined for each cow using the transrectal ultrasonography method [74]. On Day 60 of gestation, blood samples (10 mL) were obtained, 3 h after the consumption of DDGS, RUAA, or RPAA, from the jugular vein into tubes without coagulants (for serum) or with heparin (for plasma), and cows that were not pregnant were removed from the study. Plasma or serum was obtained after centrifugation at 600× *g* for 10 min. The supernatant fluid was stored at −80 °C until analyzed.

Calves from the previous pregnancy stayed with their mothers until they were weaned at 6 months of age. Those calves did not consume any supplements provided to their mothers. When the cows in the current study gave birth to new calves, gestation length, the birth weights of calves, and the number of calves born alive or dead were recorded.

### 2.2. Analyses of Hormones in Serum and of Metabolites in Plasma

Serum was analyzed for insulin and progesterone using the Mercodia Insulin ELISA kit (Uppsala, Sweden) and the Abnova Progesterone ELISA kit (Walnut, CA, USA), respectively. Amino acids in plasma were analyzed using a high-performance liquid chromatography method involving precolumn derivatization with 30 mM *o*-phthaldialdehyde [9]. Ammonia, urea, and glucose in plasma were analyzed using enzymatic methods involving glutamate dehydrogenase, urease, and hexokinase, respectively [75].

### 2.3. Statistical Analysis

Analysis of variance (ANOVA) was applied to compare the means of age and body weight across groups, whereas the chi-square test was applied to compare distributions of BCS and pasture across groups [76]. A logistic regression model was used to evaluate the effects of treatment on pregnancy rate (the number of pregnant cows/the total number of cows receiving AI) and birth rate (the number of live-born calves/the total number of cows receiving AI) [77]. Because the means and variances of all measured variables (where applicable) did not differ (*p* > 0.05) between the RUAA and RPAA groups (Supplementary Table S1) and because it is now known that extracellular Cit is not degraded in the rumen of cattle [56,57,58], data from these two groups were combined as the Cit group to increase the power of statistical analysis. For ANOVA, quantile–quantile plots on residuals and the F-test were used to test the normality and homogeneity of variances [76], respectively (*p* > 0.05), and the data were not transformed. Differences among treatment means were determined using the Student–Newman–Keuls multiple comparison method as the post hoc test in all ANOVA. Data on the plasma concentrations of metabolites and hormones in beef cows in the control and Cit groups were analyzed by the unpaired *t*-test. Probability (*p*) values ≤ 0.05 were taken to indicate statistical significance.

## 3. Results

### 3.1. Maternal Variables, Embryonic Survival, and Gestation Length

Beef cows in all the treatment groups grazed normally and appeared healthy. On the day of AI, all measured variables for the characteristics of the beef cows that produced calves in the control and Cit groups did not differ (*p* > 0.05; Table 3); and the values for all these cows (n = 34) were as follows: days postpartum, 68.4 ± 2.1 days; age, 6.73 ± 0.63 years; BW, 465.2 ± 12.1 kg; and BCS, 4.50 ± 0.16 (means ± SEM). On Days 40 and 60 of gestation, confirmed pregnancies were the same (Table 4), and ultrasound analysis showed that all cows carried a single fetus. There were no pregnancy losses in the cows between Days 40 and 60 of gestation. Gestation length did not differ (*p* > 0.05) between the control and Cit groups of cows (Table 3) and averaged 281.8 ± 1.0 days (means ± SEM, n = 34). In addition, the ratio of male to female offspring did not differ (*p* > 0.05) between these two groups of cows (Table 3). All these variables for the three treatment groups of cows that produced calves are presented in Supplemental Table S2.

Among the control, RUAA, and RPAA groups that produced calves, there was no difference in age (*p* = 0.25), body weight (*p* = 0.53), BCS (*p* = 0.61), or pasture assignment (*p* = 0.84) from the logistic regression models. Based on the results from the logistic regression models, when compared with the control group, there was no difference (*p* = 0.61) in the cow pregnancy rates between the RPAA and RUAA groups. On Days 40 and 60 of gestation, the pregnancy rates in the control and Cit groups were 25.0% and 35.2%, respectively (*p* = 0.045; Table 4). One calf was born dead in the control group, but all calves were born alive in the Cit group. The birth rates for live-born calves in the control and Cit groups were 22.2% and 35.2%, respectively (*p* = 0.040; Table 4). Data on pregnancy or birth rates for the three treatment groups of cows are shown in Supplemental Table S3. Live-born calves grew well and had no mortality within 3 months after birth.

### 3.2. Concentrations of Hormones and Metabolites in Maternal Serum or Plasma

There were no differences (*p* > 0.05) in concentrations of progesterone in maternal serum between the control and Cit groups of cows (Table 5). However, dietary supplementation with Cit increased (*p* < 0.001) the concentration of insulin in maternal serum by 82%. In addition, concentrations of Cit, Arg, ornithine, and proline in maternal plasma were 19%, 20%, 19%, and 17% greater (*p* < 0.05), respectively, in the Cit group than in the control group (Table 6). Concentrations of other amino acids, glucose, and urea in maternal plasma did not differ (*p* > 0.05) between these two groups of cows (Table 6). In contrast, dietary Cit supplementation decreased (*p* < 0.05) the concentrations of ammonia in maternal plasma by 14% (Table 6). All these variables for the three treatment groups of cows on Day 60 of gestation are summarized in Supplemental Tables S4 and S5.

## 4. Discussion

Cit (a neutral and chemically stable amino acid) is an effective precursor for the synthesis of Arg in mammals [78,79,80,81,82,83], including ruminants [18,53,84,85]. Although Cit is synthesized de novo from glutamine/glutamate and proline by the small-intestine mucosa via the pyrroline-5-carboxylate synthase and proline oxidase pathways, respectively [18,52,86], this metabolic pathway is insufficient to provide adequate amounts of Arg in gestating dams and fetuses [10,87,88,89,90,91]. There is considerable evidence that Arg plays a crucial role in embryonic survival, placental angiogenesis, and reproductive efficiency in mammals (including rats, swine, sheep, and humans) by serving as substrates for the formation of NO, polyamines, creatine, and protein [10,11,19,20,21,44,85,92,93,94,95,96,97,98,99]. Similar results have been reported for dietary supplementation with Cit to gestating rats [12,91,100,101,102,103,104,105,106], swine [107], goats [67], and sheep [61,108]. To our knowledge, there is no report of the impact of dietary supplementation with Cit or Arg on embryonic/fetal survival in beef or dairy cattle. The present study is the first to demonstrate a beneficial effect of dietary supplementation with Cit in improving pregnancy outcomes in lactating beef cattle. Several salient findings deserve additional discussions.

The pregnancy rate (25%) in the control group of lactating beef cows was relatively low (Table 4). This may be related to a number of factors, including climate, stress induced from daily handling, and the suboptimal BCS of the cow herd in a subtropical region [109,110,111,112,113], where there are usually high temperatures [e.g., a high temperature of 30.6 °C and humidity of 96% on the day of AI (1 June 2016), as well as a high temperature of 35.0 °C and a humidity of 43% on 10 July 2016)] in the Texas summer. Relatively low BSCs at the time of breeding are not uncommon for beef cattle in the southeastern United States [2,112,114]. Additionally, there was inclement weather on the day of AI with heavy thunderstorms and rain, likely causing added stress to the cows. The BCS of the cows used in this study (a mean value of 4.56) on the day of AI was slightly lower than the ideal BCS of cows at breeding that would be between 5 and 6 [114,115,116]. However, the BCS of the experimental cows did not differ between the control and Cit groups (Table 2). Of note, a low rate of pregnancy in lactating beef cattle with heat stress (e.g., 16% in AI-bred beef cows in July in Central Texas) has been reported by other investigators [117]. For AI cows, low pregnancy rates may result from many factors, including ovarian dysfunction, chromosomally abnormal or meiotically immature oocytes, impaired embryonic development, impaired implantation, embryonic/fetal death, and abortion of the fetal/placental tissue [112,118,119,120]. Of particular note, dietary supplementation with Cit (RUAA or RPAA) enhanced pregnancy rates of lactating beef cows at Day 40 or 60 of gestation from 25% to 35% and birth rates for live-born calves from 22% to 35% (Table 4). This novel finding highlights a crucial role of Cit or Arg in improving embryonic survival in cattle.

**Figure 2:**
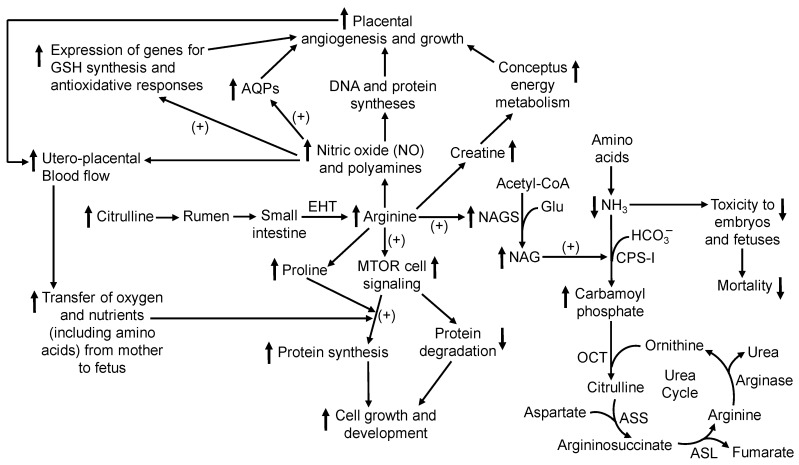
Proposed biochemical mechanisms responsible for the beneficial effect of dietary L-citrulline (Cit) supplementation in improving pregnancy outcomes in beef cows. Dietary Cit bypasses catabolism in the rumen to enter the small intestine, where it is absorbed into the portal circulation. Cit is not taken up by the liver and instead is effectively used for the synthesis of L-arginine (Arg) by extrahepatic tissues and cells (e.g., the kidneys, placentae, and endothelial cells) in gestating dams. Arg is the precursor for the formation of nitric oxide, polyamines, and creatine that are essential for placental angiogenesis and growth, uterine–umbilical blood flow, the transfer of oxygen and nutrients (e.g., amino acids, glucose, water, fatty acids, vitamins, and ions) from mother to fetus via aquaporins (AQPs), and conceptus energy metabolism. In addition, Arg stimulates the secretion of insulin from pancreatic β-cells and activates the mechanistic target of rapamycin (MTOR) signaling pathway in cells, thereby promoting the initiation and elongation of protein synthesis while inhibiting proteolysis. Furthermore, Arg increases the expression of genes for the synthesis of glutathione (GSH), as well as antioxidative and anti-inflammatory responses in maternal tissues and conceptuses. Finally, as an activator of N-acetylglutamate (NAG) synthase, which catalyzes the formation of NAG (an allosteric activator of carbamoyl phosphate synthase), Arg enhances the removal of ammonia (a metabolite that is highly toxic to the conceptus at elevated concentrations). Abbreviations: EHT, the conversion of citrulline into arginine in extra-hepatic tissues in the presence of aspartate via argininosuccinate synthase (ASS) and argininosuccinate lyase (ASL); ↑, increase or improve; ↓, decrease; “+”, activation.

The beneficial effects of the AA supplement were associated with increases in the concentrations of (a) insulin in maternal serum (Table 4) and (b) Cit and metabolically related amino acids (i.e., Arg, ornithine, and proline) in maternal plasma (Table 4) that are essential building blocks of proteins. Arg is hydrolyzed by arginase to urea and ornithine, which is metabolized to (a) proline via ornithine aminotransferase and pyrroline-5-carboxylate reductase and (b) polyamines via ornithine decarboxylase, spermidine synthase, and spermine synthase [52]. Thus, dietary Cit entered the portal circulation and served as the immediate precursor for synthesis of Arg in extrahepatic tissues. About 90% of Arg in the blood bypasses the liver but is readily taken up by extrahepatic tissues via cationic amino acid transporters (CAT1, CAT2A, CAT2B, and CAT3) in mammals [121], including ruminants [84,122,123]. Extracellular Arg stimulates (a) the synthesis of NO, polyamines, and creatine that are essential for placental angiogenesis and growth, uterine–umbilical blood flow, the transfer of water and ions from mother to fetus, and conceptus energy metabolism [124,125,126,127]; (b) MTOR signaling for the initiation and elongation of protein synthesis [19,20,21,44,128,129]; (c) the secretion of insulin from pancreatic β-cells to enhance anabolic metabolism [130,131,132]; and (d) the expression of genes for the synthesis of glutathione, as well as antioxidative and anti-inflammatory responses [133,134]. At the dose included in the supplement, rumen-protected or unprotected glutamine did not affect the concentrations of Cit or Arg in the plasma of cattle or sheep [57,58,135]. Likewise, dietary supplementation with 0.5% Cit did not influence concentrations of amino acids (including alanine, lysine, and histidine) other than Arg, ornithine, and proline (Table 5), indicating that this nutritional method did not result in an amino acid imbalance in the body. Furthermore, Arg allosterically activates *N*-acetylglutamate (NAG) synthase, the enzyme that catalyzes the formation of NAG [136]. The latter serves as an allosteric activator of carbamoyl phosphate synthase (an enzyme of the urea cycle) [137]. Thus, Arg enhances the removal of ammonia (which is highly toxic to mammalian embryos and fetuses at elevated concentrations) [138] via its conversion into urea and therefore protects the conceptus from damage by ammonia derived from both the rumen and the whole-body oxidation of amino acids [26,29]. Based on these likely actions of Arg, the biochemical mechanisms responsible for its effect in improving pregnancy outcomes in lactating beef cows are graphically summarized in Figure 2.

The corpus luteum plays a crucial role in maintaining pregnancy in mammals by producing progesterone, and Arg may modulate luteal function in ruminants [139]. In support of this view, daily intraperitoneal infusions of 40 mg Arg/kg BW/day between 40 and 140 days of gestation increased the concentration of progesterone in plasma by 8.8% [140]. In contrast, there are reports that the intravenous administration of 81 mg Arg/kg BW/day for 21–26 days did not affect the circulating levels of progesterone in either pregnant sheep between 100 and 125 days of gestation [9] or nonpregnant sheep during the estrous cycle [141]. It is possible that the design of the present study did not allow us to detect a small increase in progesterone.

Birth weight is influenced by not only the size and function of the placenta but also the provision of nutrients [1]. In the present study, Cit supplementation ended quite early during the initial period of placental development and thus likely did not influence birth weights. Although bovine placentomes begin to form as early as on Day 22 of gestation, even by Day 60 of gestation (the end of supplementation) they are usually very small and cannot be felt by palpation [2,3,4,5,6,7]. In multifetal pregnancies (e.g., swine [11] and prolific ewes [8]), improved embryonic survival is not necessarily associated with an increase in birth weight. Results of the present study indicate that this is also true for pregnant cattle carrying singletons (Table 4). A longer period of Cit supplementation may enhance both conceptus survival and birth weight in cattle, as recently reported for ewes [108].

Embryonic/fetal deaths represent the single greatest economic loss for beef cows [4,5,110]. Cows that become pregnant after the first AI, embryo transfer, or natural service are more profitable, because additional costs due to more days on feed, synchronization of estrus, AI or embryo transfer, or human labor are incurred with each unsuccessful attempt to establish pregnancy [2,69]. Additionally, with increased pregnancies resulting from the initial AI, producers can effectively shorten their breeding season, which then results in heavier weaning weights and a uniform calf crop to increase their marketability [142].

A successful pregnancy in beef or dairy cows is currently estimated to be worth USD 750 or even >USD 1000 per calf [143]. Based on the cost of Cit (USD 10/kg) [144] and the daily use of 0.07 kg/day for 60 days (i.e., a total of 4.2 kg Cit/cow), the total expense for feeding one cow would be USD 42. For an operation with 1000 beef cows, the net income would be USD 62,250 and USD 131,750, respectively, at the price of USD 750 and USD 1250 per calf (Table 7). Additional benefits that are not included in the margin of profit calculation include reductions in management and labor costs, improvements in herd health, an increase in cow numbers, and the prospect of higher fertility in the next pregnancy. A distinct advantage of the use of Cit over Arg is that the half-life of Cit in the maternal plasma of pregnant mammals including ruminants is 1.5 h, which is much longer than that for Arg (0.76 h) [145,146]. Thus, dietary supplementation with Cit is more effective than Arg in increasing Arg availability in both the mother and the fetus [53]. In addition, as for Arg [8,12,147], maternal dietary supplementation with Cit can program offspring for improved postnatal growth, survival, and health, possibly due to enhanced prenatal development of the pancreas, skeletal muscle, and cell signaling pathways [102,108]. Based on the results of the present study, Cit added directly as a supplement to diets without any encapsulation bypasses the rumen in ruminants (including gestating beef cattle) and is more affordable for use by producers. This eliminates the need for the encapsulation of Cit or Arg via expensive engineering techniques as a rumen-protected product [148] and their impractical administration via intravenous [8,38,39,40] or intragastric infusions [149,150,151] in production settings. Because the price of feed-grade Cit without encapsulation will be substantially lower than that for the human-food grade product [152], such a simple nutrition-based management method to increase embryonic survival will have an enormous impact on the global beef industry. These findings also have important implications for enhancing both milk production and fertility in lactating dairy cows [68,153,154,155], because they also have very low pregnancy rates, e.g., 16% in the Southern U.S. (e.g., Central Texas) in the summer [110]. Finally, our dietary Cit supplementation is likely applicable to other mammals, including sows [14,156], humans [157], sheep [108], and goats [66,67] to improve health and productivity while alleviating or preventing fetal programming of metabolic syndrome in adulthood [111,158,159].

## 5. Conclusions

The cause of the low pregnancy rate in the control group was likely caused by multiple factors, including a lower than ideal BCS of the cattle, high ambient temperatures, and the untimely thunderstorm that occurred on the day of AI. Dietary supplementation with Cit in either a rumen-protected or unprotected form to lactating beef cattle between Days 1 and 60 of gestation increased concentrations of Cit, Arg, ornithine, proline, and insulin in maternal blood, decreased concentrations of ammonia in maternal plasma, and improved embryonic/fetal survival. This simple and cost-effective method for dietary supplementation with Cit is expected to reduce early pregnancy losses and increase reproductive efficiency and profitability in cattle and other livestock enterprises. Large-scale experiments are warranted to optimize supplemental doses and evaluate economic returns from the nutritional treatment.

## Figures and Tables

**Table 1:** Characteristics of all beef cows that received artificial insemination (AI) ^1^.

Variable	Control (n = 36)	RUAA ^2^ (n = 35)	RPAA ^2^ (n = 36)	*p*-Value
Days postpartum on the day of AI (days)	67.1 ± 1.5	68.5 ± 1.8	66.9 ± 1.9	0.817
Age on the day of AI (years)	5.86 ± 0.21	6.86 ± 0.64	5.97 ± 0.46	0.261
Body weight on the day of AI (kg)	457.3 ± 11.6	473.8 ± 14.0	459.4 ± 12.8	0.568
Body condition score on the day of AI	4.66 ± 0.10	4.54 ± 0.12	4.48 ± 0.10	0.479

^1^ Values are means ± SEM, with the number of beef cows being indicated within the parentheses. ^2^ The supplemental dose of Cit was equivalent to 0.5% of the estimated daily feed intake (14 kg dry matter) of a cow on pasture. Control, no citrulline supplementation; RPAA, rumen-protected citrulline; RUAA, unprotected citrulline.

**Table 2:** Content of nutrients in the aerial parts of pasture grasses grazed by beef cows ^1^.

Nutrient	Pasture A	Pasture B	*p*-Value
Dry matter (DM; %)	31.46 ± 0.38	31.60 ± 0.28	0.774
Water (%)	68.54 ± 0.38	68.4 ± 0.28	0.774
Ash, % of DM	7.92 ± 0.07	7.88 ± 0.06	0.676
Organic matter, % of DM	92.08 ± 0.07	92.12 ± 0.06	0.676
Neutral detergent fiber (NDF, % of DM)	70.46 ± 0.35	70.58 ± 0.37	0.820
Acid detergent fiber (ADF, % of DM)	35.12 ± 0.40	35.42 ± 0.49	0.648
Hemicellulose ^2^ (% of DM)	35.34 ± 0.47	35.16 ± 0.44	0.787
Crude protein (CP, % of DM)	13.92 ± 0.17	14.02 ± 0.09	0.617
Crude fat (CF, % of DM)	2.50 ± 0.07	2.44 ± 0.06	0.533
Soluble carbohydrates ^3^ (% of DM)	5.20 ± 0.21	5.08 ± 0.32	0.762

^1^ Values are means ± SEM, n = 5. The aerial parts of grasses were collected from five areas (four corners and the center) of each pasture in September 2016. ^2^ Calculated as the difference between NDF and ADF. ^3^ Calculated as organic matter − (ADF + CP + CF).

**Table 3:** Characteristics of beef cows that produced calves, and the sexes of the calves ^1^.

Variable	Control (n = 9)	0.5% Cit (n = 25)	*p*-Value
Days postpartum on the day of AI (days)	67.9 ± 3.9	68.6 ± 2.6	0.889
Age of cows on the day of AI (years)	5.89 ± 0.45	7.13 ± 0.89	0.421
Body weight of cows on the day of AI (kg)	463.8 ± 18.9	465.8 ± 15.0	0.943
Body condition score on the day of AI	4.5 ± 0.16	4.5 ± 0.14	1.00
Gestation length (days)	283.9 ± 1.7	281.2 ± 1.0	0.177
Number (and %) of male newborn calves	4 (44.4%)	11 (45.8%)	0.981
Number (and %) of female newborn calves	5 (55.6%)	14 (54.2%)	0.981

^1^ Values are means ± SEM, with the numbers of cows in the parentheses. From Day 1 to Day 60 of gestation, cows were individually fed daily either 0.84 kg of dried distillers grains with solubles (DDGS; control) or 0.56 kg of DDGS plus 0.28 kg of an amino acid supplement [containing 0.07 kg of L-citrulline (Cit)]. The supplemental dose of Cit was equivalent to 0.5% of the estimated daily intake of 14 kg dry matter from pasture. AI, artificial insemination.

**Table 4:** Calving data for beef cows following artificial insemination ^1^.

Treatment Group	Number of Cows Receiving AI	Confirmed Pregnancies (or %) from AI Service on Day 40 ^2^	Number of Cows Reaching Term	Number of Live Calves at Birth	Birth Rate for Live- Born Calves (%)	Birth Weight of Live-Born Calves (kg) [Means ± SEM]	Number of Calves Born Dead
Control	36	9 (25.0%)	9	8	22.2	29.0 ± 1.3	1
0.5% Cit	71	25 (35.2%)	25	25	35.2	26.9 ± 0.71	0
*p*-Value	---	0.045	---	---	0.040	0.167	---

^1^ All calves born were singles. From Day 1 to Day 60 of gestation, cows were individually fed daily either 0.84 kg of dried distillers grains with solubles (DDGS; control) or 0.56 kg of DDGS plus 0.28 kg of an amino acid supplement [containing 0.07 kg of L-citrulline (Cit)]. The supplemental dose of Cit was equivalent to 0.5% of the estimated daily intake of 14 kg dry matter from pasture. Ultrasound analysis showed that all cows carried singletons on Days 40 and 60 of gestation. There was no pregnancy loss in all groups of cows between Days 40 and 60 of gestation. ^2^ The number within the parentheses refers to a pregnancy rate (the number of pregnant cows/the total number of cows receiving AI). Pregnancy or birth rates were analyzed based on the logistic regression models.

**Table 5:** Concentrations of hormones in the serum of gestating beef cows on Day 60 of gestation ^1^.

Hormone	Control (n = 9)	0.5% Cit (n = 25)	*p*-Value
Progesterone (ng/mL)	1.99 ± 0.12	2.01 ± 0.08	0.896
Insulin (µIU/mL)	132 ± 16	240 ± 21	0.006

^1^ Values are means ± SEM, with the numbers of cows in parentheses. From Day 1 to Day 60 of gestation, cows were individually fed daily either 0.84 kg of dried distillers grains with solubles (DDGS; control) or 0.56 kg of DDGS plus 0.28 kg of an amino acid supplement [containing 0.07 kg of L-citrulline (Cit)]. The supplemental dose of Cit was equivalent to 0.5% of the estimated daily intake of 14 kg dry matter from pasture. Serum samples were obtained from beef cows on Day 60 of gestation for analyses.

**Table 6:** Concentrations of amino acids, ammonia, urea, and glucose in the plasma of gestating beef cows on Day 60 of gestation ^1^.

Variable	Control	0.5% Cit	*p*-Value
	(n = 9)	(n = 25)	
Alanine (nmol/mL)	235 ± 8.7	237 ± 7.1	0.878
β-Alanine (nmol/mL)	16 ± 2.0	18 ± 1.1	0.368
Arginine (nmol/mL)	80 ± 3.6	96 ± 2.7	0.003
Asparagine (nmol/mL)	32 ± 1.9	35 ± 1.6	0.311
Aspartate (nmol/mL)	9.3 ± 0.5	9.5 ± 0.4	0.787
Citrulline (nmol/mL)	57 ± 2.3	68 ± 2.0	0.005
Cysteine ^2^ (nmol/mL)	103 ± 4.8	107 ± 3.9	0.579
Glutamate (nmol/mL)	60 ± 2.6	61 ± 2.3	0.812
Glutamine (nmol/mL)	328 ± 16	339 ± 8.9	0.538
Glycine (nmol/mL)	196 ± 7.8	204 ± 5.5	0.443
Histidine (nmol/mL)	42 ± 2.2	43 ± 1.1	0.660
Isoleucine (nmol/mL)	103 ± 4.9	107 ± 3.5	0.546
Leucine (nmol/mL)	128 ± 5.4	132 ± 4.1	0.602
Lysine (nmol/mL)	97 ± 5.0	100 ± 3.3	0.636
Methionine (nmol/mL)	27 ± 1.0	29 ± 1.0	0.270
Ornithine (nmol/mL)	70 ± 3.2	83 ± 2.9	0.018
Phenylalanine (nmol/mL)	50 ± 2.2	53 ± 1.5	0.297
Proline (nmol/mL)	142 ± 6.4	166 ± 5.1	0.015
Serine (nmol/mL)	57 ± 2.8	60 ± 2.1	0.447
Taurine (nmol/mL)	26 ± 1.4	27 ± 1.0	0.597
Threonine (nmol/mL)	58 ± 2.5	61 ± 2.1	0.439
Tryptophan (nmol/mL)	52 ± 2.9	55 ± 1.9	0.413
Tyrosine (nmol/mL)	58 ± 2.3	62 ± 1.7	0.890
Valine (nmol/mL)	196 ± 12	205 ± 7.2	0.525
Ammonia ^3^ (nmol/mL)	87 ± 4.1	75 ± 2.6	0.022
Urea (nmol/mL)	6051 ± 368	6047 ± 332	0.995
Glucose (nmol/mL)	3472 ± 303	3461 ± 251	0.983

^1^ Values are means ± SEM, with the numbers of cows in parentheses. From Day 1 to Day 60 of gestation, cows were individually fed daily either 0.84 kg of dried distillers grains with solubles (DDGS; control) or 0.56 kg of DDGS plus 0.28 kg of an amino acid supplement [containing 0.07 kg of L-citrulline (Cit)]. The supplemental dose of Cit was equivalent to 0.5% of the estimated daily intake of 14 kg dry matter from pasture. Plasma samples were obtained from beef cows on Day 60 of gestation for analyses. ^2^ Free cysteine + ½ cystine. ^3^ NH_4_^+^ + NH_3_.

**Table 7:** Economic return from dietary supplementation with L-citrulline to lactating beef cows.

1000 Beef Cows	Live-Born Calves	Income (USD)	Supplement Cost (USD)	Net Income Gain (USD)
USD 750/calf				
Control	222	166,500	0	166,500
L-Citrulline ^1^	361	270,750	42,000	228,750
Difference	139	104,250	42,000	62,250
USD 1250/calf				
Control	222	277,500	0	277,500
L-Citrulline ^1^	361	451,250	42,000	409,250
Difference	139	173,750	42,000	131,750

^1^ 4.2 kg of L-citrulline is supplemented to a cow for 60 days. The cost of L-citrulline is USD 10/kg.

## Data Availability

All data are contained within this article.

## Supplementary Materials

The following supporting information can be downloaded at: https://www.mdpi.com/article/10.3390/ani15162398/s1, Table S1: Probability values for comparisons between RUAA and RPAA groups; Table S2: Characteristics of beef cows that produced calves; Table S3: Calving data for beef cows following artificial insemination (AI); Table S4: Concentrations of hormones in the serum of gestating beef cows; Table S5: Concentrations of amino acids, ammonia, urea, and glucose in the plasma of gestating beef cows.
