# Supplementary material for: Dietary Supplementation with L-Citrulline Between Days 1 and 60 of Gestation Enhances Embryonic Survival in Lactating Beef Cows
Source: Animals (Basel). 2025 Aug 15;15(16):2398. doi: 10.3390/ani15162398 (PMC12383036; doi:10.3390/ani15162398)
Supplement: Supplementary file 1 [file animals-15-02398-s001.zip › animals-3770237-supplementary.pdf]

Supplementary Table S1. Probability values for comparisons between RUAA and RPAA groups [1].

**Item**	**Cow's BCS**	**Cow's Body Weight**	**Cow's Age**	**Pregnancy or Birth Rate**
Mean	0.461	0.435	0.257	0.876
Variance	0.466	0.755	0.071	Not applicable

[1] The numbers of cows in the RUAA and RPAA groups that produced calves are 12 and 13, respectively. BCS, body condition score.

Supplementary Table S2. Characteristics of beef cows that produced calves [1].

Variable	Control (n = 9)	RUAA [2] (n = 12)	RPAA [2] (n = 13)	*p*-Value
Days postpartum on the day of AI (days)	67.9 ± 3.9	70.9 ± 3.6	66.3 ± 3.6	0.653
Age on the day of AI (years)	5.89 ± 0.45	7.18 ± 1.21	7.08 ± 1.29	0.712
Body weight on the day of AI (kg)	463.8 ± 18.9	471.0 ± 28.0	460.7 ± 14.3	0.936
Body condition score on the day of AI	4.5 ± 0.16	4.5 ± 0.18	4.5 ± 0.21	1.00
Gestation length (days)	283.9 ± 1.7	280.9 ± 1.6	281.4 ± 1.2	0.376
Number (and %) of male newborn calves	4 (44.4%)	5 (41.7%)	6 (46.2%)	0.985
Number (and %) of female newborn calves	5 (55.6%)	7 (58.3%)	7 (53.8%)	0.985

[1] Values are means ± SEM, with the number of beef cows being indicated within the parentheses. Data were analyzed by one-way ANOVA. [2] The supplemental dose of Cit was equivalent to 0.5% of the estimated daily feed intake (14 kg of dry matter) of a cow on pasture. Control, no citrulline supplementation; RPAA, rumen-protected citrulline); RUAA, unprotected citrulline.

Supplementary Table S3. Calving data for beef cows following artificial insemination (AI) [1].

**Treatment Group**	**Number of Cows Receiving AI**	**Confirmed Pregnancies (or %) from AI Service on Day 40** [2]	**Number of Cows Reaching Term**	**Number of Live Calves at Birth**	**Birth Rate for Live-Born Calves (%)**	**Birth Weight of Live-Born Calves (kg) [Means ± SEM]**	**Number of Calves Born Dead**
Control	36	9 (25.0%)	9	8	22.2	29.0 ± 1.3	1
RUAA [3]	35	12 (34.3%)	12	12	34.3	25.9 ± 1.1	0
RPAA [3]	36	13 (36.1%)	13	13	36.1	27.8 ± 0.9	0
p-Value	---	0.091	---	---	0.082	0.165	---

[1] All calves born were singles. [2] The number within the parenthesis refers to a pregnancy rate (the number of pregnant cows/the total number of cows receiving artificial insemination). [3] The supplemental dose of Cit was equivalent to 0.5% of the estimated daily feed intake (14 kg of dry matter) of a cow on pasture. There was no pregnancy loss in all groups of cows between Days 40 and 60 of gestation. Pregnancy or birth rates were analyzed based on the logistic regression models. Control, no citrulline supplementation; RPAA, rumen-protected citrulline; RUAA, unprotected citrulline.

Supplementary Table S4. Concentrations of hormones in the serum of gestating beef cows [1].

**Hormone**	**Control (n = 9)**	**RUAA** [2] **(n = 12)**	**RPAA** [2] **(n = 13)**	***p*-Value**
Progesterone (ng/mL)	1.99 ± 0.12	2.00 ± 0.11	2.01 ± 0.12	0.996
Insulin (µIU/mL)	132 ± 16 [b]	259 ± 30 [a]	221 ± 29 [a]	< 0.001

[1] Serum samples were obtained from beef cows on Day 60 of gestation. Values are means ± SEM, with the number of beef cows being indicated within the parentheses. [2] The supplemental dose of Cit was equivalent to 0.5% of the estimated daily feed intake (14 kg of dry matter) of a cow on pasture. [a-b]: Within a row, means not sharing the same superscript letter differ ($p < 0.05$). Control, no citrulline supplementation; RPAA, rumen-protected citrulline; RUAA, unprotected citrulline.

Supplementary Table S5. Concentrations of amino acids, ammonia, urea, and glucose in the plasma of gestating beef cows [1].

AA	Control (n = 9)	RUAA [2] (n = 12)	RPAA [2] (n = 13)	*p*-Value
Aspartate	9.3 ± 0.5	9.6 ± 0.7	9.4 ± 0.5	0.938
Glutamate	60 ± 2.6	60 ± 3.2	61 ± 3.1	0.963
Asparagine	32 ± 1.9	35 ± 2.2	34 ± 2.2	0.535
Serine	57 ± 2.8	59 ± 3.2	60 ± 2.8	0.946
Glutamine	328 ± 16	336 ± 12	341 ± 13	0.804
Histidine	42 ± 2.2	42 ± 1.8	44 ± 1.4	0.635
Glycine	196 ± 7.8	201 ± 7.2	206 ± 8.2	0.684
Threonine	58 ± 2.5	60 ± 2.1	61 ± 2.2	0.662
Citrulline	57 ± 2.3 [b]	67 ± 2.8 [a]	68 ± 3.1 [a]	0.030
Arginine	80 ± 3.6 [b]	95 ± 3.8 [a]	97 ± 3.7 [a]	0.009
β-Alanine	16 ± 2.0	18 ± 1.4	17 ± 1.7	0.727
Taurine	26 ± 1.4	27 ± 1.6	27 ± 1.6	0.890
Alanine	235 ± 8.7	239 ± 10	234 ± 9.6	0.923
Tyrosine	58 ± 2.3	61 ± 2.2	62 ± 2.6	0.524
Tryptophan	52 ± 2.9	54 ± 2.4	56 ± 2.8	0.602
Methionine	27 ± 1.0	29 ± 1.5	28 ± 1.2	0.587
Valine	196 ± 12	207 ± 11	203 ± 9.2	0.783
Phenylalanine	50 ± 2.2	53 ± 2.4	52 ± 1.8	0.641
Isoleucine	103 ± 4.9	106 ± 4.2	108 ± 5.5	0.792
Leucine	128 ± 5.4	130 ± 4.9	133 ± 6.6	0.852
Ornithine	70 ± 3.2 [b]	82 ± 3.8 [a]	84 ± 4.0 [a]	0.033
Lysine	97 ± 5.0	99 ± 4.7	101 ± 4.4	0.831
Proline	142 ± 6.4 [b]	167 ± 7.1 [a]	165 ± 7.3 [a]	0.032
Cysteine [3]	103 ± 4.8	106 ± 5.2	107 ± 5.6	0.874
Ammonia [4]	87 ± 4.1 [a]	75 ± 3.5 [b]	74 ± 3.8 [b]	0.042
Urea	6051 ± 368	6019 ± 437	6075 ± 492	0.985
Glucose	3472 ± 303	3402 ± 328	3520 ± 376	0.968

[1] Plasma samples were obtained from beef cows on Day 60 of gestation. Values, expressed as nmol/mL, are means ± SEM, with the number of beef cows being indicated within the parentheses. [2] The supplemental dose of Cit was equivalent to 0.5% of the estimated daily feed intake (14 kg of dry matter) of a cow on pasture. [3] Free cysteine + ½ cystine. [4] NH_4^+ + NH_3. [a-b]: Within a row, means not sharing the same superscript letter differ ($p < 0.05$). Control, no citrulline supplementation; RPAA, rumen-protected citrulline; RUAA, unprotected citrulline.
